# The LINC00922 aggravates ovarian cancer progression via sponging miR-361-3p

**DOI:** 10.1186/s13048-021-00828-7

**Published:** 2021-06-11

**Authors:** Liping Wang, Chenchen Ren, Yajuan Xu, Li Yang, Yannan Chen, Yuanhang Zhu

**Affiliations:** Department of Obstetrics and Gynecology, Third Affiliated Hospital, Zhengzhou University, Henan Province, Zhengzhou, 450052 China

**Keywords:** Ovarian Cancer, LINC00922, MiR-361-3p

## Abstract

**Background:**

Long noncoding RNA (lncRNA) LINC00922 has been reported to promote tumorigenesis of lung and breast cancer. However, the functions and mechanisms of LINC00922 in ovarian cancer (OC) remain unclarified. The current study aims to clarify the detailed functions and underlying mechanisms of LINC00922 in the progression of OC.

**Methods:**

LINC00922 expression in OC tissues and cells was identified by a comprehensive strategy of data miming, computational biology and quantitative real-time polymerase chain reaction (RT-qPCR) experiment. In vitro CCK-8, wound healing, transwell invasion, western blotting and in vivo tumorigenesis assays LINC00922 were conducted to evaluate the functions of LINC00992. Subsequently, bioinformatics technology and dual luciferase reporter assay were performed to confirm the between miR-361-3p and LINC00922 or CLDN1. Finally, rescue experiments were performed to confirm whether LINC00922 effect functions of OC cells through regulation of miR-361-3p.

**Results:**

LINC00922 was significantly upregulated in OC tissues and cell lines, which is significantly positively corelated with the poor prognosis of patients with OC. LINC00922 knockdown inhibited proliferation and tumorigenesis of OC cells in vitro and vivo. In addition, LINC00922 knockdown suppressed migration, invasion, and EMT of OC cells in vitro. Mechanically, LINC00922 could competitively bind with miR-361-3p to relieve the repressive effect of miR-361-3p on its target gene CLDN1 in OC cells. In addition, silencing miR-361-3p promoted OC cell proliferation, migration, invasion, EMT and Wnt/β-catenin signaling, while LINC00922 knockdown inhibited Wnt/β-catenin signaling by upregulating miR-361-3p. Rescue experiments revealed that LINC00922 knockdown inhibited OC cell proliferation, migration, invasion and EMT by regulating miR-361-3p.

**Conclusion:**

This study suggested that LINC00922 could competitively bind with miR-361-3p to promote the CLDN1 expression and activate Wnt/β-catenin signaling in OC progression, which providing a promising therapeutically target for OC.

**Supplementary Information:**

The online version contains supplementary material available at 10.1186/s13048-021-00828-7.

## Introduction

Ovarian cancer (OC), a frequently prevalent malignant tumor, is the primary cause of cancer-related death in women worldwide [1, 2]. In the United States, 21, 750 new cases of OC are reported in 2020. Among them, around 13, 940 mortalities were reported annually [1]. With advancements in the field of oncology diagnosis in the last decade, a 5-year survival rate of patients with OC has increased by approximately 90% [3]. However, clinical outcomes of patients with advanced OC remain unfavorable, with less than 6% of the 5-year survival rate [4]. Hence, it is very important to completely explore the OC mechanism at the molecular level.

Recent studies have focused on exploring the functions and molecular mechanisms of long non-coding RNAs (lncRNAs) in cancer [5, 6]. Numerous studies have revealed that lncRNAs played important roles in regulating behaviors of cancer cells physiologically and pathologically [7]. During the genesis and progression of cancer, cellular pathways that suppress and induce cancer are mostly regulated by lncRNAs by direct or indirect effects [8]. Recent evidences have demonstrated that lncRNAs were dysregulated in OC and were involved in the progression of OC [9, 10].

MicroRNAs (miRNAs) are defined as single-stranded, highly stable, and endogenous types of RNA molecules consisting of nucleotides [11]. Binding of miRNAs to the 3′-UTRs (3′-untranslated regions) completely or incompletely in their target genes, and negatively regulate the gene expression. By this way, miRNAs decrease the translation and hence promote the degradation of mRNAs [12]. It has been revealed that miRNAs were involved in various malignancies during the development of OC [13]. In the past few years, several evidences have determined cross-modulation between miRNAs and lncRNAs in human cancers [13, 14]. As competing endogenous RNAs (ceRNAs), lncRNAs can perform their function to regulate the mRNA expression via binding in a competitive manner with shared miRNAs [9, 15]. Therefore, the exploration of significant roles of lncRNAs provides new insight of the diagnosis and therapy for OC.

In this study, we found that LINC00922 was not only upregulated in OC tissues and cells, but also closely related to the poor prognosis of OC patients. Functional experiments showed that LINC00922 knockdown inhibited the malignant behavior of ovarian cancer cells in vitro and in vivo. Mechanically, we found that LINC00922 knockdown inhibited the expression of CLDN1 and Wnt/β-catenin signaling activation by sponging miR-361-3p, thus restraining the progression of OC.

## Material and methods

### Data collection

The LncRNA and mRNA expression profiles of OC dataset were sourced from Gene Expression Omnibus (GEO) database (accession number: GSE74440) and The Cancer Genome Atlas/Genotype-Tissue Expression (TCGA/GTEx). The miRNA expression profile dataset was obtained from GEO database (accession number: GSE119055) and TCGA/GETx. The data analysis was performed with R software using edgeR package. The threshold set for significant different genes was |log2 fold change|≥ 1 and *P*-value < 0.05. The data for survival analysis were obtained from TCGA and a log rank *P*-value < 0.05 was considered to be statistically significant. The data for the correlation analysis were obtained from TCGA ovarian cancer data set and *P*-value < 0.05 was considered to be statistically significant.

### Cell lines and cell culture

Ovarian cancer cell lines (ES-2, SKOV-3 and A2780) and normal human ovary cell line (IOSE-80) were purchased from Chinese Academy of Sciences (Shanghai, China). RPMI-1640 medium (Gibco, Grand Island, New York, USA) consist of 10% FBS (Gibco) and 1% P/S was used for culturing the ovarian cells and was incubated with 5% CO_2_ at 37 °C.

### Cell transfection

The LINC00922 small hairpin RNA (sh-linc00922), miR-361-3p mimics, miR-361-3p inhibitors and their negative controls (sh-NC、NC mimics and NC inhibitor) were purchased from RiboBio (Guangzhou, China). Sequences of sh-linc00922, miR-361-3p mimic and miR-361–3 inhibitor are shown in Table 1. Consequently, the transfection of sh-linc00922, miR-361–3 mimics, miR-361–3 inhibitor, or their negative controls (sh-NC、NC mimics and NC inhibitor) were performed using Lipofectamine 2000 reagent (Invitrogen, Carlsbad, CA, USA) according to the manufacturer’s instructions. After transfection, the quantitative real-time polymerase chain reaction (RT-qPCR) was performed to verify the transfection efficiency.

**Table 1:** Sequences of LINC00922 shRNA, miR-361-3p mimic and miR-361-3p inhibitor were used in the study

genes	Sequence (5’-3’)
miR-361-3p mimics	UCCCCCAGGUGUGAUUCUGAUUU
miR-361-3p inhibitor	AGGGGUCCACACUAAGACUAAA
LINC00922 shRNA-1	CCGGCCTGCACCTACAGATCTACACCTCGAGGTGTAGA TCTGTAGGTGCAGGTTTTTG
LINC00922 shRNA-2	CCGGGAACCTACATTCACAACATAACTCGAGTTATGTTG TGAATGTAGGTTCTTTTTG
LINC00922 shRNA-3	CCGGTGCAGGAAGTGTTCATCTAAGCTCGAGCTTAGAT GAACACTTCCTGCATTTTTG

### Fluorescence in Situ Hybridization (FISH)

FISH assay was performed to detect the subcellular localization of LINC00922 in ovarian cancer cells according to previous studies [16, 17]. Briefly, cells were fixed in 4% formaldehyde for 10 min at room temperature. Cells were permeabilized in PBS containing 0.5% Triton X-100 for 5 min at 4 °C, then washed in PBS 3 × 5 min. Hybridization was carried out with specific Cy3-labeled LINC00922 probe (RiboBio, Guangzhou, China) in a moist chamber at 37 °C in the dark overnight according to the protocol provided by the manufacturer. All images were visualized under a confocal microscope (Zeiss).

### Nuclear & cytoplasmic RNA fractionation

The nuclear and cytoplasmic RNA fractionation was isolated with cytoplasmic & nuclear RNA Purification Kit (Norgen Biotek, Canada) according to manufacturer’s protocol. Subsequently, RT-qPCR was used to measure the distribution of lncRNA in ovarian cancer cells. GAPDH was used as a loading control for the cytoplasmic gene expression, and U6 small nuclear expression was used as a loading control for the nuclear gene expression in the current study.

### RT-qPCR

Total RNA from the cells was extracted using the TRIzol reagent (Invitrogen, Carlsbad, CA, USA) according to the manufacturer’s instructions. After the extraction process has completed, the quality and concentration of total RNA was determined with a Nanodrop instrument (IMPLEN GmbH, Munich, Germany). Reverse transcription and PCR of lncRNA and mRNA were conducted using the PrimeScript™ RT reagent Kit with gDNA Eraser (Takara, Dalian, China) and TB Green® *Premix Ex Taq*™ II (Takara, Dalian, China) with GAPDH as an internal reference. Mir-X™ miRNA FirstStrand Synthesis and TB Green® RT-qPCR User Manual (Takara, Dalian, China) was employed for miRNAs with U6 as an internal control. All reactions were performed in an Applied Biosystems 7500 Fast Real-Time PCR system (Applied Biosystems, Foster City, USA), and relative expression of genes was analyzed using the 2^−ΔΔCt^ method. The experiment was performed with triplicate. All primer sequences used in this study were listed in the Table 2.

**Table 2:** Primer sequences for RT-qPCR of lncRNAs**,** miRNAs and mRNAs were used in the study

Genes	Sequence (5’-3’)
miR-361-3p	Forward primer, GAGTCCCCCAGGTGTGATTC Reverse primer, GTCGTATCCAGTGCGTGTC
LINC00922	Forward primer, TCCAGGCATGTCATCTTCACTC Reverse primer, CCACCTAGCTGTCTGTCTTCATT
LINC01711	Forward primer, CGTCACCGAGAGCCTCTTATG Reverse primer, AATCACCACTGGCTGAGAACC
DANT2	Forward primer, CAGAAGATGGAGGGAGGGATG Reverse primer, CGACTACCTCTGATCTGCGTT
SPATA41	Forward primer, AACTGTGACAAGTGACCATCCT Reverse primer, GCCTCTTCTCTGAGCGTTCT
CLDN1	Forward primer, CCCAGTCAATGCCAGGTACG Reverse primer, CAAAGTAGGGCACCTCCCAG
U6	Forward primer, CTCGCTTCGGCAGCACATATACT Reverse primer, ACGCTTCACGAATTTGCGTGTC
GAPDH	Forward primer, CTCCTCTGACTTCAACAGCGAC Reverse primer, TAGAAGATGAAAAGAGTTGTCAGGG

### Cell Counting Kit-8 (CCK-8) assay

Cell proliferation was detected by CCK-8 assay. In brief, the cells were seeded in 96-well plates and the each well was seeded with a cell density of 2.0 × 10^4^/mL. After seeding the cells, 10 μL CCK-8 (Dojindo, Kumamoto, Japan) was added into all the each well at specific time points followed by incubation for 2 h at 37 °C. Finally, the absorbance was measured at 450 nm by a microplate reader (Thermo Fisher Scientific, Waltham, MA, USA). The absorbance value was also used to plot the viability curve. The experiment was performed with triplicate.

### Cell Migration Ability

The migration ability of cells was quantified by wound-healing assay. In brief, cells were cultured in 6-well plates (1.0 × 10^5^ cells per well). With the help of the light microscope, the cell confluency was examined prior to wound scratches. The representative images of cell migration were observed and photographed under optical microscopy (Leica, Germany) at 0 and 24 h after injury. The cells migration distance was analyzed using Image J software (National Institutes of Health, Bethesda, MD). The experiment was performed with triplicate.

### Cell Invasion Ability

Transwell 24-well Boyden chamber (Corning, USA) with 8 μm a pore size was used for the cell invasion with Matrigel assay according to the manufacturer’s protocol. Initially, the plates were coated with Corning Matrigel matrix (Corning Cat. No. 354234) using a volume of 200–300 μg/ml. After 24 h, 200 μl of culture medium containing 1.0 × 10^5^ cells were plated in the upper chamber, while each bottom chamber was plated with 500 μl of culture medium containing 10% FBS and allowed to incubate for 24 h at 37 °C. The cells were then fixed with 4% paraformaldehyde for 30 min and stained with a 0.1% solution of crystal violet. A ZEISS Axio Imager.Z2 microscope was used to calculate the invaded cells.

### Bioinformatics Analysis

LncBase Predicted v.2 online database (http://carolina.imis.athena-innovation.gr/) was used for prediction of the LINC00922-targeted microRNA. The StarBase version 3.0 (http://starbase.sysu.edu.cn/) or miRDB (http://mirdb.org/index.html) was applied for prediction of interaction between mRNAs and microRNAs, and the intersection of the two software predictions were identified as the potential targets of miRNAs. The correlations of LINC00922-miRNAs, miRNAs-mRNAs and LINC00922-mRNAs pairs were analyzed using Pearson’s correlation coefficient. The correlation analysis was performed using corrplot R package. The visualization was made with ggpubr package in R software. The *P*-value < 0.05 was considered as significant correlation. The Kaplan–Meier Plotter tool was used to determine the association between lncRNAs and the prognosis of patient with OC. Moreover, competitive endogenous RNA (ceRNA) network of LINC00922 / miRNA / mRNA was constructed by using the Cytoscape v.3.6.1 software.

### Dual luciferase reporter assay

The dual-luciferase reporter system (Promega) was employed for gene detection according to the manufacturer's instructions. LINC00922 and CLDN1 3’UTR wild and mutant plasmids were constructed using pmirGLO Dual-Luciferase vector. Ovarian cancer cells were co-transfected with wild or mutant vectors and mimics using Lipofectamine^TM^2000 reagent (Invitrogen, Carlsbad, CA, USA). After co-transfected for 48 h, Dual Luciferase Reporter Assay System (Promega) were used to detect the firefly luciferase and Renilla luciferase activity, and Renilla luciferase activity was used for normalizing firefly luciferase activity.

### Nuclear protein extraction

The nuclear protein extraction was performed using the Nuclear and Cytoplasmic Protein Extraction Kit (Beyotime, Shanghai, China) according to the protocol of manufacturer, and then subjected to western blotting analysis using ant-β-catenin. LaminB1 were used as loading controls for nuclear protein fraction.

### Western blotting

Total protein was extracted from cells using pre-cooled RIPA buffer (Beyotime, Shanghai) containing protease and phosphatase inhibitors (Thermo Scientific, USA), and then Bicinchoninic Acid Protein Assay Kit (Thermo Scientific, USA) was used for protein quantification. An equal amount of protein samples was separated by SDS-PAGE (Beyotime, Shanghai) and then transferred to a membrane of polyvinylidene fluoride (PVDF) (Millipore, Billerica, MA, USA). After being blocked by skim milk (5%), the membrane was further nurtured with the corresponding primary antibodies at 4 °C overnight and then the HRP-conjugated secondary antibodies for two hours at room temperature. The blots were visualized with the ECL detection reagent (Pierce) using the Chemi-Doc imaging system (Bio-Rad Laboratories, Inc.). Protein expression levels were quantified by densitometry using Image J software (National Institutes of Health, Bethesda, MD) and normalized to β-actin. Western blotting was performed using the following primary antibodies: PCNA (1:1000; #ab29, Abcam, England), E-cadherin (1:1000; # 3195, CST, USA), Vimentin (1:1000; #ab92547, Abcam, England), CLDN1 (1:1000, # ab211737, Abcam, England), Wnt (1:1000, # ab15251, Abcam, England), β-catenin (1:1000; # 8480S, CST, USA), LaminB1 (1:1000; # 13435S, CST, USA) and β-actin (# cw0096m, CWBiotech, China). Horseradish peroxidase (HRP)-conjugated anti-mouse or anti-rabbit IgG was used as the secondary antibody.

### Animal experiments

All animal experiments were approved by the Institutional Animal Care and Use Committee of the Third Affiliated Hospital of Zhengzhou University. A total of 12 female BALB/c nude mice (aged 6 ~ 8 weeks old, SPF) were purchased from the Beijing Vital River Laboratory Animal Technology Co. Ltd. (Beijing, China). The all mice were randomly divided into two grouped (*N* = 6 per group), and then the mice were subcutaneously injected the left flank with suspension of SKOV-3 cells (1.0 × 10^6^ cells) with or without silencing LINC00922(sh-linc00922 and sh-NC)in 200μL of PBS. Tumor sizes were measured with a caliper every 7 days to analyze tumor growth. The tumor volume was estimated with the formula V = (length × width2)/2. The mice were euthanized after four weeks, the tumors were removed and weighted.

### Statistical analysis

SPSS software (SPSS, Inc. version 18.0) was used for all the statistical analyses of the current study, and images were plotted using GraphPad Prism 6.0 (GraphPad Software, CA, USA). The paired or unpaired Student’s *t*-test was used for comparison between two groups and one-way ANOVA for more than two groups. All results have been conferred as mean ± SEM. *P*-value < 0.05 were considered statistically significant.

## Results

### LINC00922 is upregulated in ovarian cancer and correlated with poor prognosis

**Fig. 1:**
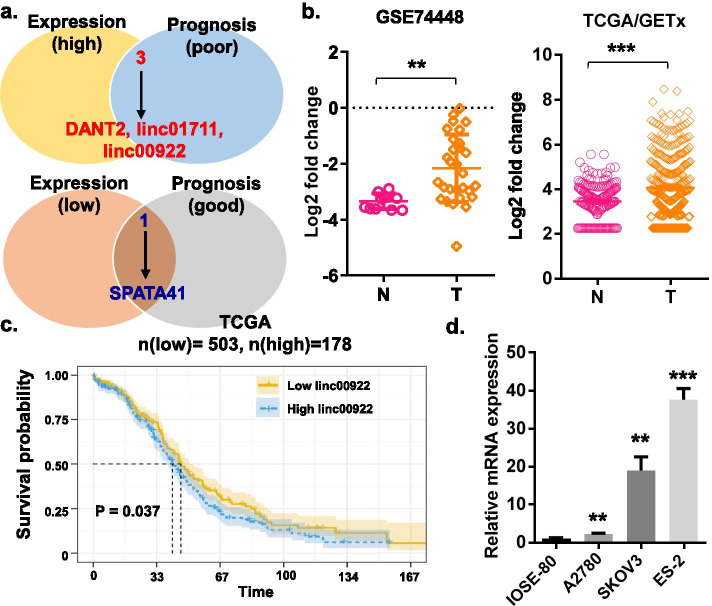
LINC00922 was upregulated in OC. **a** Venn diagram showing critical lncRNAs regulating OC progression. **b** The expression level of LINC00922 in that of OC tissues along with normal ovarian tissues based on GSE74448 and TCGA/GETx datasets. **c** Kaplan–Meier curve was plotted for the analyses of patients' survival rate on the basis of LINC00922 expression. **d** RT-qPCR was performed for ascertaining the expression of LINC00922 in three OC cell lines namely A2780, SKOV3, and ES-2 along with a human ovarian cell line named IOSE-80. **P < 0.01; ***P < 0.001

We initially identified the critical lncRNAs regulating OC progression based on GSE74448 and TCGA/GETx OC data sets (Figs. S1-S2 and Fig. 1). Combined differential expression and survival analysis, we found that DANT2, LINC01711, and LINC00922 were significantly upregulated in OC tissues and positively correlated with poor prognosis of patients with OC. APATA41 was downregulated in OC tissues and gave a prediction of a good prognosis (Fig. 1a-c, and Fig. S2a-b). Further, three OC cell lines (A2780, SKOV-3 and ES-2) and normal ovarian epithelial cells (IOSE-80) were used to validate the relative expression of these 4 selected lncRNAs by RT-qPCR. Compared with that in normal ovarian epithelial cells (IOSE-80), LINC00922 expression in three OC cell lines was significantly higher (Fig. 1d). However, LINC01711 and SPATA41 were up-regulated only in SKOV3 or ES-2, DANT2 showed no significant difference in A2780 and SKOV3 while down-regulated in ES-2, which was inconsistent with the previous analysis results (Fig. S2c). The aforementioned data illustrated that LINC00922 was upregulated in OC and that high expression of LINC00922 was associated with poor outcomes in OC.

### Downregulation of LINC00922 inhibits cell proliferation and tumor growth in ovarian cancer

**Fig. 2:**
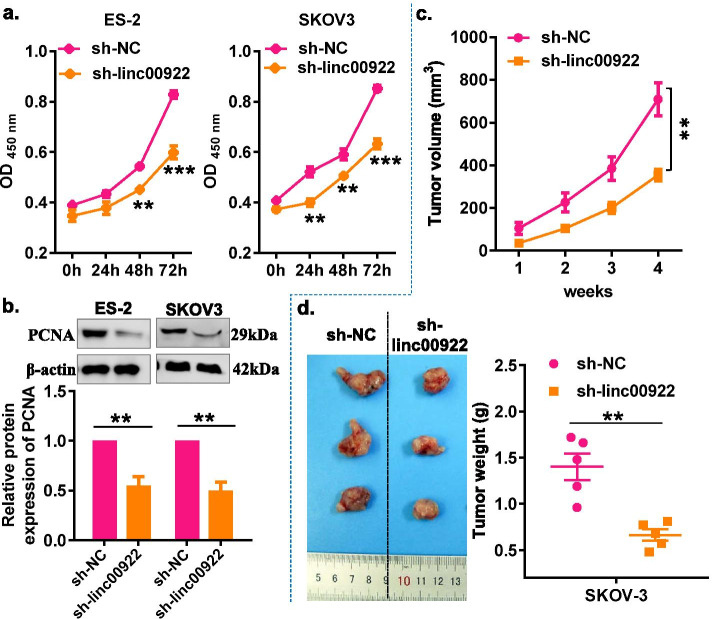
LINC00922 knockdown inhibited the proliferation and tumorigenesis of ovarian cancer cells in vitro and in vivo. **a** CCK-8 assay analysis showed proliferation of OC cells was inhibited via LINC00922 knockdown. **b** Western Blot showed the protein expression of PCNA in that of OC cells was inhibited via LINC00922 knockdown. **c** Growth curves of xenograft tumors of each group. **d** Representative images and weight of xenograft tumors of each group. sh-linc00922, LINC00922 small hairpin RNA. sh-NC, corresponding negative control of sh-linc00922. **P < 0.01, ***P < 0.001

To investigate the biological function of LINC00922 in OC cells, the sh-linc00922 was applied to knock down LINC00922, and the knockdown efficiency was confirmed by RT-qPCR. The results showed that sh-linc00922#3 was the most efficient in reducing LINC00922 expression (Fig. S3). Therefore, sh-linc00922#3 (sh-linc00922) was selected for subsequent experiment. Growth curves performed by the CCK-8 assay revealed that LINC00922 knockdown significantly inhibited the proliferation viability of ES-2 and SKOV3 cells (Fig. 2a). Similarly, the LINC00922 knockdown markedly inhibited the protein expression of PCNA in ES-2 and SKOV-3 cells (Fig. 2b). Furthermore, a subcutaneous xenograft model was used to validate the effect of LINC00922 on tumor growth in vivo. Consistent with in vitro results, LINC00922 knockdown significantly inhibited tumor weight and tumor volume of SKOV-3 cells compared with those in the control group (Fig. 2c, d). These experiments suggested that LINC00922 knockdown could inhibit proliferation of OC cells both in vitro and vivo.

### Downregulation of LINC00922 inhibits cell migration and invasion in ovarian cancer

**Fig. 3:**
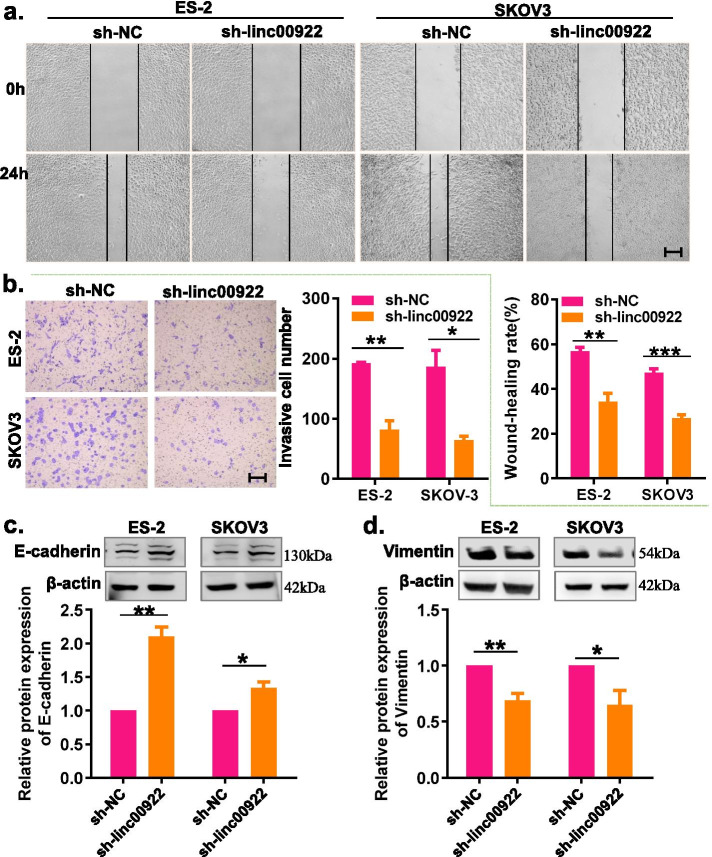
LINC00922 knockdown inhibited migration, invasion, and EMT of ovarian cancer cells. **a** Migration capacities was carried out by healing assays after transfected with indicated vectors (sh-linc00922 or sh-NC). **b** Invasion capacities was carried out by transwell assays after transfected with indicated vectors (sh-linc00922 or sh-NC). **c**, **d** LINC00922 knockdown promoted the expression of PCNA and decreased Vimentin expression in ovarian cancer cells determined by performing western-blot assay. Scale bar: 50 μm. sh-linc00922, LINC00922 small hairpin RNA. sh-NC, corresponding negative control of sh-linc00922. *P < 0.05, **P < 0.01, ***P < 0.001

We then investigated the role of LINC00922 in the migration and invasion of OC cells through the wound healing assay and transwell invasive assay. The results showed that LINC00922 knockdown significantly suppressed the migration and invasion of SKOV3 and ES-2 cells (Fig. 3a, b). Furthermore, we evaluated whether LINC00922 knockdown effected on epithelial-mesenchymal transition (EMT) of OC cells by detecting the protein expression of EMT-related markers. Western blot analysis demonstrated that LINC00922 knockdown could promote the protein expression of epithelial markers (E-cadherin) and inhibit the protein expression of mesenchymal marker (Vimentin) (Fig. 3c, d), indicating that LINC00922 could regulate the EMT process to modulate OC progression. Taken together, these results suggested that LINC00922 act as an oncogenic gene which promoted the migration and invasion of OC.

### LINC00922 regulates CLDN1 expression via competitively binding to miR-361-3p in ovarian cancer

**Fig. 4:**
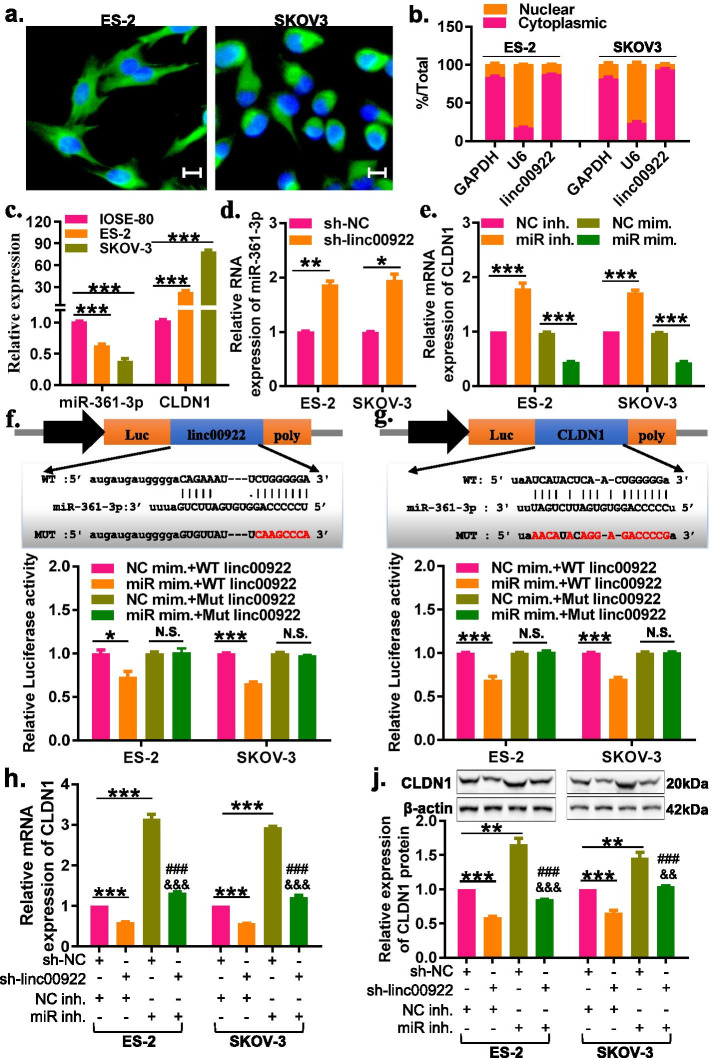
LINC00922 binds to miR-361-3p positively regulating the expression of CLDN1. **a**, **b** Cellular localization of LINC00922 in ovarian cancer cells was identified by RNA FISH and of cytoplasmic and nuclear RNA fractions assays. **c** The expression of miR-361-3p and CLDN1 was detected by RT-qPCR. **d** RT-qPCR assay demonstrated the expression of miR-361-3p in ovarian cancer cells through transfection with sh-linc00922. **e** The expression of CLDN1 were identified by performing RT-qPCR in that of ovarian cancer cells through transfection with either miR-361-3p mimics or miR-361-3p inhibitor. **f** Schematic illustration of LINC00922-WT and LINC00922-MUT luciferase reporter vectors, and the relative luciferase activities were detected in OC cells after transfection with LINC00922-WT or LINC00922-Mut and miR-361-3p mimics or NC mimics, respectively. **g** Schematic illustration of CLDN1-WT and CLDN1-MUT luciferase reporter vectors, and the relative luciferase activities were detected in OC cells after transfection with CLDN1-WT or CLDN1-MUT and miR-361-3p mimics or NC mimics, respectively. **h**, **j** The mRNA and protein expression of CLDN1 were identified in ovarian cancer cells after that of co-transfection with sh-linc00922 along with either miR-361-3p inhibitor or NC inhibitor. sh-linc00922, LINC00922 small hairpin RNA. sh-NC, corresponding negative control of sh-linc00922. miR inh., miR-361-3p inhibitor. NC inh., corresponding negative control of miR-361-3p inhibitor. *P < 0.05, **P < 0.01, ***P < 0.001 vs. sh-linc00922 + NC inh. ^###^*P* < 0.001 vs. sh-linc00922 + NC inh. ^&&^*P* < 0.01, ^&&&^*P* < 0.001 vs. sh-NC + miR inhibitor

To elucidate the underlying mechanism of LINC00922 in OC, we conducted the fluorescence in situ hybridization (FISH) assay and cytoplasmic and nuclear RNA fractions assay to identity the LINC00922 location in ES-2 and SKOV3 cells. The FISHanalysis showed that LINC00922 was predominantly located in the cytoplasm of ES-2 and SKOV3 cells (Fig. 4a), which was further confirmed by cytoplasmic and nuclear RNA fractions assay (Fig. 4b), suggesting that LINC00922 acts as a ceRNA in that of OC. Therefore, we constructed the LINC000922-miRNAs-mRNAs ceRNA network based on the comprehensive analysis of the data miming and bioinformatics analysis. The potential binding miRNAs (285 miRNAs) of LINC00922 were predicted by the LncBase Predicted v.2 online database, of which 11 miRNAs were significantly down-regulated in ovarian cancer tissues through analysis of miRNA-OC dataset GSE119055 (Fig. S4a, and Table 3). These 11 miRNAs were considered as the potential binding miRNAs of LINC00922 in ovarian cancer**.** Subsequently, the potential target genes of these 11 miRNAs were predicted in miRDB and StarBase. A total of 4908 genes were observed to be intersected, of which 307 genes were significantly up-regulated in ovarian cancer tissues through analysis of miRNA-OC dataset GSE7448 (Fig. S4b, and Table. 4). These 307 genes were considered as potential target genes of miRNAs in ovarian cancer. Then, a LINC00922/miRNA/mRNA ceRNAs network containing LINC00922, 11 miRNAs and 307 mRNAs was constructed by using the Cytoscape v.3.6.1 software (Fig. S4c). LncRNAs perform specific functions by competitively bind to miRNAs and eliminate miRNA-mediated degradation of direct targets.[18]. Based on this hypothesis, there is a negative correlation between miRNAs and lncRNAs or mRNAs, whilst there is a positive correlation between mRNAs and lncRNAs. Here, we used TCGA OC data to analyze the correlation of mRNA/miRNA or miRNA/lncRNA or mRNA/lncRNA in the established network (Fig. S4c). The results showed that LINC00922/miRNAs pairs (LINC00922/miR-361-3p and LINC00922/miR-29b-2-5p), miR-361-3p or miR-29b-2-5p/mRNAs pairs (miR-361-3p/CLDN1, miR-361-3p/PODXL and miR-29–2-5p/SNX30), and LINC00922/mRNAs pairs(LINC00922/CLDN1)were consistent with ceRNAs mechanism (Fig. S4d). Based on the three levels into consideration, we constructed a new LINC00922/miR-361-3p/CLDN1 triple sub-network (Fig. S4e), which may be involved in the progression of OC.

**Table 3:** The changes in the expression levels of 11 miRNAs in ovarian cancer

lncRNA	miRNA	logFC	*P*.Value
LINC00922	hsa-miR-874-3p	-1.42	3.09E-02
	hsa-miR-628-5p	-1.02	3.71E-02
	hsa-miR-497-5p	-2.34	1.81E-02
	hsa-miR-495-3p	-3.92	1.77E-02
	hsa-miR-361-3p	-2.31	1.15E-02
	hsa-miR-29b-2-5p	-1.59	3.94E-02
	hsa-miR-299-5p	-4.00	2.81E-02
	hsa-miR-214-5p	-3.49	3.15E-02
	hsa-miR-204-5p	-4.50	4.39E-03
	hsa-miR-195-5p	-2.34	1.09E-02
	hsa-miR-128-3p	-1.31	3.97E-02

**Table 4:** The changes in the expression levels of 307 mRNAs in ovarian cancer

miRNA	mRNA	logFC	*P*.Value	mRNA	logFC	*P*.Value	mRNA	logFC	*P*.Value	mRNA	logFC	*P*.Value
hsa-miR-874-3p	RRM2	3.66	8.66E-09	BTN3A2	1.50	1.31E-03	SYNGR2	1.69	1.09E-08	THRB	1.30	3.95E-05
	VEGFA	3.13	9.41E-14	SORT1	1.48	1.78E-08	IL12A	1.69	4.98E-05	IKBKB	1.23	9.93E-10
	PASK	2.54	1.73E-11	LPCAT4	1.47	3.48E-09	BCL7A	1.67	2.15E-09	KCNK9	1.19	7.74E-06
	PARVG	2.38	7.25E-08	ENTPD8	1.40	1.00E-04	HAPLN1	1.64	1.55E-08	GFAP	1.19	4.09E-03
	ZNF710	2.03	3.67E-12	DNMT3B	1.37	1.21E-10	FARP1	1.50	1.99E-07	MARCH9	1.17	8.42E-04
	SLC11A1	1.82	1.21E-08	PFKFB4	1.35	3.15E-05	LSM14B	1.09	1.00E-04	PPP1R16B	1.17	3.99E-03
	GRIP1	1.78	3.06E-07	SLC5A3	1.33	3.12E-06	SEC14L2	1.08	1.21E-03	SMG1	1.13	5.03E-06
	BDH1	1.71	1.22E-05	DYRK2	1.32	3.16E-06	ZNF682	1.08	2.89E-03	SGSM2	1.09	9.89E-05
	SLC35E2B	1.06	3.45E-05	SIM2	1.00	2.33E-03	ADGRE2	2.25	3.95E-13	SLIT2	1.21	2.75E-03
hsa-miR-628-5p	VCAN	3.41	1.97E-06	SLC5A1	1.44	2.66E-03	GRIP1	1.78	3.06E-07	RAG1	1.21	6.32E-06
	KATNAL2	3.21	7.30E-11	ST6GAL1	1.34	1.94E-05	PHYHIPL	1.46	4.70E-03	HYDIN	1.14	1.81E-04
	CCDC146	2.34	4.54E-07	PDLIM1	1.25	9.75E-05	CD80	1.04	9.27E-05	TMEM185B	1.07	6.10E-04
hsa-miR-497-5p	RNF125	6.22	4.28E-20	GABRE	2.52	1.07E-09	NAT8L	2.87	4.15E-07	PCDHA13	1.97	9.80E-06
	MFAP5	4.36	2.85E-07	SEL1L3	2.50	9.11E-09	ANLN	2.68	4.44E-07	UNC5B	1.97	4.77E-06
	RNF43	4.33	1.20E-10	CCDC88C	2.48	1.99E-13	SOX6	2.68	1.95E-06	HAS2	1.92	6.68E-05
	RNF183	4.12	1.02E-12	NOL4L	2.30	7.84E-12	ESRP1	2.63	6.20E-12	PCDHA5	1.88	6.84E-07
	LGR5	4.00	1.05E-06	PLEKHH1	2.25	5.86E-09	PLEKHB2	1.22	1.26E-02	ZDHHC23	1.82	2.96E-08
	TFCP2L1	3.92	6.77E-10	RAPGEFL1	2.19	4.98E-12	SLIT2	1.21	2.75E-03	CHFR	1.72	7.06E-08
	THSD4	3.78	4.97E-09	SH3BP2	2.19	1.71E-11	NPAS3	1.17	5.27E-04	FAM189B	1.68	7.90E-09
	RSPO3	3.30	4.22E-04	XDH	2.16	5.76E-06	HMGA2	1.15	5.75E-07	BCL7A	1.67	2.15E-09
	AHNAK2	3.29	2.26E-09	BHLHE41	2.15	6.06E-07	PDPR	1.15	2.13E-07	LHPP	1.66	4.42E-09
	VEGFA	3.13	9.41E-14	SGPL1	2.11	2.81E-10	SMURF2	1.14	9.45E-04	ANKRD33B	1.64	2.56E-06
	MYB	3.03	2.13E-11	PGM2L1	2.10	7.21E-06	MOV10	1.13	3.73E-08	LCP1	1.60	2.98E-03
	NUAK2	3.00	6.93E-17	MYO5B	2.08	2.14E-07	TMEM74B	1.09	9.11E-04	KCNK15	1.57	5.43E-04
	PTPN3	2.89	1.57E-14	CBX2	2.05	6.53E-07	ODF2L	1.08	1.85E-06	FSTL4	1.55	6.18E-04
	PISD	1.02	1.14E-04	ATXN7L2	1.44	7.42E-05	PWWP2B	1.08	7.63E-05	SLC36A1	1.54	7.08E-06
	CHD6	1.01	1.19E-02	SPSB4	1.43	3.50E-03	GATAD2A	1.05	1.90E-05	RAB4B	1.54	1.04E-07
	ZFHX3	1.01	1.35E-03	AREL1	1.42	2.55E-06	LRRK1	1.04	1.53E-04	CYB561	1.50	1.17E-04
	AK4	1.34	2.51E-04	RELT	1.38	1.03E-04	CD80	1.04	9.27E-05	TFAP2A	1.50	5.75E-05
	CCDC6	1.29	1.16E-03	MCU	1.38	4.82E-07	ZNF367	1.03	2.12E-03	RNF213	1.48	2.01E-10
	RAB11FIP1	1.27	1.62E-05	TLL1	1.37	4.73E-03	EML6	1.02	7.55E-03	CLUH	1.44	2.35E-09
	CADM1	1.26	9.74E-06	PFKFB4	1.35	3.15E-05	ZNF691	1.26	6.72E-05	KLHL18	1.34	1.29E-09
	IKBKB	1.23	9.93E-10	AMOT	1.34	6.27E-04	FMN1	2.71	3.06E-14	JAG2	1.94	1.36E-05
hsa-miR-495-3p	KLHL14	6.01	1.84E-21	SLC2A1	2.49	1.58E-06	ANLN	2.68	4.44E-07	SASH1	1.94	3.76E-05
	OVOL2	5.65	9.81E-22	PPP1R1C	2.37	1.97E-06	SOX6	2.68	1.95E-06	TMTC2	1.90	6.27E-09
	DCDC2	5.42	3.31E-16	ARHGAP26	2.29	1.72E-13	TDRD5	2.63	2.55E-06	BAIAP2	1.87	6.27E-12
	BMP7	5.35	9.94E-12	GEN1	2.27	7.90E-12	CDH1	2.54	1.14E-07	KIAA1324	1.83	5.07E-03
	GRHL2	5.01	3.42E-17	TNC	2.23	3.99E-05	SDC3	1.34	3.06E-05	SHROOM3	1.77	4.90E-11
	SCEL	3.39	4.62E-08	RCOR1	2.22	1.98E-06	SHOX2	1.34	4.50E-03	DAPP1	1.72	5.78E-08
	SHISA9	3.23	3.53E-06	SSX2IP	2.18	1.57E-09	DNMT3A	1.33	1.32E-07	SPATA6L	1.71	1.88E-04
	BCL11A	3.17	2.45E-10	SLC35F2	2.10	1.25E-06	GABBR2	1.32	1.03E-03	PREX1	1.67	1.01E-04
	VEGFA	3.13	9.41E-14	HCAR1	2.10	5.87E-05	NFE2L3	1.31	7.96E-06	ABHD17C	1.67	1.66E-04
	MECOM	2.93	1.68E-15	PGM2L1	2.10	7.21E-06	CASP2	1.31	8.83E-09	PLCH1	1.63	1.06E-05
	CDK1	2.88	4.90E-10	BCL2L1	2.08	3.87E-07	THRB	1.30	3.95E-05	KLK10	1.62	2.88E-04
	CDH6	2.82	1.42E-05	BCAT1	2.08	2.37E-04	SLC44A5	1.29	8.68E-03	EPHB2	1.58	3.86E-06
	ZFP64	1.18	3.24E-06	TMEM185B	1.07	6.10E-04	CCDC6	1.29	1.16E-03	NDC1	1.57	2.74E-05
	MET	1.18	9.47E-07	TNFRSF21	1.06	6.96E-03	PELI1	1.28	6.89E-04	KLF5	1.56	1.69E-09
	NPAS3	1.17	5.27E-04	AIFM2	1.05	9.28E-05	MSR1	1.28	1.94E-03	DNAH7	1.55	3.65E-04
	SLC30A7	1.16	5.35E-07	STYK1	1.04	5.37E-04	DAW1	1.27	1.29E-03	TIAM2	1.54	2.44E-08
	CDK16	1.16	5.15E-05	GPD1	1.04	5.49E-05	CADM1	1.26	9.74E-06	UBN1	1.51	1.68E-06
	TNRC18	1.16	8.48E-05	NR6A1	1.03	8.00E-03	SLFN13	1.26	4.66E-06	FARP1	1.50	1.99E-07
	RDH10	1.14	4.00E-04	ZNF250	1.03	7.64E-05	ZNF691	1.26	6.72E-05	DDIAS	1.47	1.46E-08
	BAK1	1.14	5.40E-04	SNX30	1.01	7.99E-08	NHSL1	1.26	4.06E-05	KDM2A	1.46	1.28E-05
	UPF3A	1.12	1.09E-02	CHD6	1.01	1.19E-02	AK5	1.25	4.74E-03	ITGB5	1.44	1.56E-04
	CFAP44	1.12	2.21E-03	ZFHX3	1.01	1.35E-03	SLC38A10	1.25	6.73E-07	SPSB4	1.43	3.50E-03
	SP1	1.11	8.55E-04	PPIL2	1.00	1.23E-05	SLAIN1	1.22	2.61E-04	AREL1	1.42	2.55E-06
	CASP8	1.10	2.26E-05	CEP76	1.07	5.61E-04	UBE2Z	1.22	1.43E-09	KMO	1.40	9.08E-04
	GPD2	1.21	8.81E-05	MCU	1.38	4.82E-07	TMEM156	1.21	1.16E-04	MAK	1.40	6.11E-06
	FRAS1	1.19	3.13E-04	EDN1	1.35	2.37E-03						
hsa-miR-361-3p	CLDN1	4.27	1.53E-09	VASH1	1.64	1.25E-04	BAIAP2	1.87	6.27E-12	CLUH	1.44	2.35E-09
	FAM72D	3.09	2.00E-12	TMEM104	1.61	3.61E-07	XG	1.72	5.57E-04	GABBR2	1.32	1.03E-03
	ALDH3B2	2.70	1.19E-07	RNFT2	1.61	8.29E-06	PODXL	1.70	1.91E-11	NHSL1	1.26	4.06E-05
	TRAF2	2.35	6.20E-13	SETD6	1.59	4.22E-08	IGSF3	1.69	4.16E-09	SMG1	1.13	5.03E-06
	POLQ	2.30	4.61E-15	SLC36A1	1.54	7.08E-06	SLC43A2	1.01	1.61E-07	CELSR3	1.13	1.54E-07
	SIM2	1.00	2.33E-03	UGT8	1.11	2.92E-03						
hsa-miR-29b-2-5p	DCDC2	5.42	3.31E-16	CCDC88C	2.48	1.99E-13	VEGFA	3.13	9.41E-14	HAPLN1	1.64	1.55E-08
	CAPN12	3.66	2.28E-10	BCAT1	2.08	2.37E-04	SOX6	2.68	1.95E-06	KLHL18	1.34	1.29E-09
	ADGRG2	3.24	1.77E-07	VANGL1	1.94	6.30E-08	ZFHX3	1.01	1.35E-03	SNX30	1.01	7.99E-08
	COL23A1	3.48	3.87E-08	DGAT2	2.02	9.61E-06	SMC4	2.37	2.73E-09	SEZ6L2	1.87	2.35E-05
	FMN1	2.71	3.06E-14	MMP12	1.96	3.14E-03	PRR11	2.32	7.79E-14	PHYHIPL	1.46	4.70E-03
	ERBB3	2.53	6.77E-16	VANGL1	1.94	6.30E-08	TLL1	1.37	4.73E-03	CLUH	1.44	2.35E-09
	TNFAIP8	2.46	1.89E-07	TMTC2	1.90	6.27E-09	SP1	1.11	8.55E-04	VKORC1L1	1.44	2.63E-08
	MPP3	1.41	4.03E-04									
hsa-miR-214-5p	SLC28A3	2.78	2.47E-07	SLC44A5	1.29	8.68E-03	ANKRD65	2.06	9.36E-07	CREB5	1.24	2.15E-06
	SLC2A1	2.49	1.58E-06	POLH	1.28	8.98E-09	KLF5	1.56	1.69E-09	DNM2	1.22	1.10E-10
	TRAF2	2.35	6.20E-13	SLFN13	1.26	4.66E-06	KDM2A	1.46	1.28E-05	TMEM156	1.21	1.16E-04
	BHLHE41	2.15	6.06E-07	ZNF691	1.26	6.72E-05	IQSEC3	1.36	4.45E-04	FRAS1	1.19	3.13E-04
	TAF10	1.07	2.01E-05	HMGA2	1.15	5.75E-07						
hsa-miR-204-5p	FAM3B	4.28	1.09E-08	PDPR	1.15	2.13E-07	PLCH1	1.63	1.06E-05	VANGL1	1.94	6.30E-08
	THSD4	3.78	4.97E-09	CELSR3	1.13	1.54E-07	RNFT2	1.61	8.29E-06	HAS2	1.92	6.68E-05
	RSPO3	3.30	4.22E-04	DOCK8	1.13	8.04E-05	EPHB2	1.58	3.86E-06	TMTC2	1.90	6.27E-09
	ARNTL2	3.18	4.96E-10	SP1	1.11	8.55E-04	PROSER1	1.55	5.91E-11	ALPL	1.89	4.94E-07
	BCL11A	3.17	2.45E-10	SASS6	1.04	1.86E-06	FARP1	1.50	1.99E-07	KMT5A	1.88	7.89E-09
	CDK1	2.88	4.90E-10	EDEM1	1.04	3.52E-05	TFAP2A	1.50	5.75E-05	CCDC120	1.81	2.81E-08
	ERBB3	2.53	6.77E-16	STYK1	1.04	5.37E-04	KDM2A	1.46	1.28E-05	CCDC40	1.74	1.45E-07
	PPP1R1C	2.37	1.97E-06	ZNF367	1.03	2.12E-03	GTPBP2	1.45	2.49E-06	ORAI2	1.68	8.96E-07
	PRR11	2.32	7.79E-14	SLC43A2	1.01	1.61E-07	AP1S3	1.43	1.26E-05	ARHGAP30	1.66	2.24E-06
	ARHGAP26	2.29	1.72E-13	CMKLR1	1.01	5.70E-03	GET4	1.42	8.99E-08	NOX4	1.65	1.99E-03
	CDC7	2.28	9.71E-09	ZFHX3	1.01	1.35E-03	NOS1AP	1.41	2.44E-09	GPBP1L1	1.30	2.83E-12
	BORA	2.27	2.13E-08	ESCO2	2.12	6.94E-09	HTT	1.39	9.33E-08	AMOT	1.34	6.27E-04
	RCOR1	2.22	1.98E-06	SGPL1	2.11	2.81E-10	TMEM108	1.38	2.11E-05	AK4	1.34	2.51E-04
	VASH1	1.64	1.25E-04	ZMYND8	2.07	4.61E-10	RELT	1.38	1.03E-04	GABBR2	1.32	1.03E-03
	HAPLN1	1.64	1.55E-08	LILRB2	1.96	7.09E-08	TANC1	1.38	8.10E-10	THRB	1.30	3.95E-05
	SLA2	1.00	5.71E-04	NAT8L	2.87	4.15E-07	SLC17A5	1.38	3.23E-03	CTNNBIP1	1.34	1.29E-05
hsa-miR-195-5p	RNF125	6.22	4.28E-20	ANLN	2.68	4.44E-07	TLL1	1.37	4.73E-03	MYO5B	2.08	2.14E-07
	VTCN1	5.69	2.78E-13	SOX6	2.68	1.95E-06	PFKFB4	1.35	3.15E-05	CBX2	2.05	6.53E-07
	MFAP5	4.36	2.85E-07	ESRP1	2.63	6.20E-12	KLHL18	1.34	1.29E-09	PCDHA13	1.97	9.80E-06
	RNF43	4.33	1.20E-10	GABRE	2.52	1.07E-09	AMOT	1.34	6.27E-04	UNC5B	1.97	4.77E-06
	RNF183	4.12	1.02E-12	SEL1L3	2.50	9.11E-09	AK4	1.34	2.51E-04	HAS2	1.92	6.68E-05
	LGR5	4.00	1.05E-06	CCDC88C	2.48	1.99E-13	DYRK2	1.32	3.16E-06	PCDHA5	1.88	6.84E-07
	TFCP2L1	3.92	6.77E-10	NOL4L	2.30	7.84E-12	THRB	1.30	3.95E-05	ZDHHC23	1.82	2.96E-08
	THSD4	3.78	4.97E-09	GEN1	2.27	7.90E-12	CCDC6	1.29	1.16E-03	CSF1	1.76	7.98E-06
	RSPO3	3.30	4.22E-04	PLEKHH1	2.25	5.86E-09	RAB11FIP1	1.27	1.62E-05	FAM189B	1.68	7.90E-09
	AHNAK2	3.29	2.26E-09	RAPGEFL1	2.19	4.98E-12	CADM1	1.26	9.74E-06	BCL7A	1.67	2.15E-09
	VEGFA	3.13	9.41E-14	SH3BP2	2.19	1.71E-11	ZNF691	1.26	6.72E-05	LHPP	1.66	4.42E-09
	MYB	3.03	2.13E-11	XDH	2.16	5.76E-06	IKBKB	1.23	9.93E-10	ANKRD33B	1.64	2.56E-06
	NUAK2	3.00	6.93E-17	BHLHE41	2.15	6.06E-07	PLEKHB2	1.22	1.26E-02	LCP1	1.60	2.98E-03
	PTPN3	2.89	1.57E-14	SGPL1	2.11	2.81E-10	SLIT2	1.21	2.75E-03	FSTL4	1.55	6.18E-04
	MCU	1.38	4.82E-07	PGM2L1	2.10	7.21E-06	NPAS3	1.17	5.27E-04	SLC36A1	1.54	7.08E-06
	PWWP2B	1.08	7.63E-05	SPSB4	1.43	3.50E-03	HMGA2	1.15	5.75E-07	RAB4B	1.54	1.04E-07
	GATAD2A	1.05	1.90E-05	AP1S3	1.43	1.26E-05	PDPR	1.15	2.13E-07	CYB561	1.50	1.17E-04
	LRRK1	1.04	1.53E-04	AREL1	1.42	2.55E-06	SMURF2	1.14	9.45E-04	TFAP2A	1.50	5.75E-05
	CD80	1.04	9.27E-05	RELT	1.38	1.03E-04	MOV10	1.13	3.73E-08	RNF213	1.48	2.01E-10
	ZNF367	1.03	2.12E-03	CHD6	1.01	1.19E-02	TMEM74B	1.09	9.11E-04	CLUH	1.44	2.35E-09
	EML6	1.02	7.55E-03	ZFHX3	1.01	1.35E-03	ODF2L	1.08	1.85E-06	ATXN7L2	1.44	7.42E-05
	PISD	1.02	1.14E-04									
hsa-miR-128-3p	LIX1	5.57	1.80E-09	CHST11	1.26	1.19E-04	SASH1	1.94	3.76E-05	ABCB9	1.13	1.99E-05
	EHF	3.61	4.83E-14	HOXA9	1.25	3.94E-03	TMTC2	1.90	6.27E-09	SP1	1.11	8.55E-04
	OSBPL10	3.15	9.89E-12	IGF2BP3	1.21	2.07E-04	LIMK1	1.90	4.91E-09	UGT8	1.11	2.92E-03
	CDH6	2.82	1.42E-05	EPB41	1.21	2.14E-04	KMT5A	1.88	7.89E-09	COL3A1	1.10	3.14E-02
	AGRN	2.72	1.01E-12	GPD2	1.21	8.81E-05	GRIP1	1.78	3.06E-07	VAV3	1.08	7.41E-05
	LMNB1	2.66	8.70E-08	HIVEP3	1.19	4.79E-04	CSF1	1.76	7.98E-06	CEP76	1.07	5.61E-04
	CA12	2.46	2.06E-06	MET	1.18	9.47E-07	EPB41L4A	1.75	7.24E-08	GATAD2A	1.05	1.90E-05
	PLEKHH1	2.25	5.86E-09	DOT1L	1.18	1.69E-04	IGSF3	1.69	4.16E-09	STYK1	1.04	5.37E-04
	RCOR1	2.22	1.98E-06	NPAS3	1.17	5.27E-04	ABHD17C	1.67	1.66E-04	AGO3	1.01	6.56E-06
	ALDH1L2	2.16	1.17E-09	TNRC18	1.16	8.48E-05	HAPLN1	1.64	1.55E-08	ZFHX3	1.01	1.35E-03
	PGM2L1	2.10	7.21E-06	SMURF2	1.14	9.45E-04	PLCH1	1.63	1.06E-05	GABBR2	1.32	1.03E-03
	SEMA4D	2.07	4.25E-08	ARHGEF11	1.14	5.49E-09	FN1	1.60	7.92E-03	CDH24	1.30	1.85E-04
	VANGL1	1.94	6.30E-08	MCF2L	1.14	4.02E-07	RGS1	1.59	7.89E-03	THRB	1.30	3.95E-05
	HTT	1.39	9.33E-08	CADM1	1.26	9.74E-06	EPHB2	1.58	3.86E-06	SMPD4	1.30	1.06E-06
	RELT	1.38	1.03E-04	AK4	1.34	2.51E-04	SLC36A1	1.54	7.08E-06	IFITM10	1.29	4.41E-06
	PFKFB4	1.35	3.15E-05	SLC5A3	1.33	3.12E-06	ABCA1	1.41	1.28E-06	RAB11FIP1	1.27	1.62E-05
	RCOR3	1.34	3.17E-05									

Subsequently, to validate whether LINC00922 could sponge miR-361-5p to inhibit the CLDN1 expression in OC cells. The RT-qPCR analysis revealed that miR-361-3p expression in SKOV-3 and ES-2 cells was significantly downregulated compared with this in IOSE-80, while CLDN1 expression was significantly upregulation (Fig, 4c). Moreover, LINC00922 knockdown significantly increased miR-361-3p expression (Fig. 4d). Overexpression of miR-361-3p could markedly decrease the mRNA and protein expression of CLDN1, whereas downregulation of miR-361-3p could significantly increase the mRNA and protein expression of CLDN1 in SKOV-3 and ES-2 cells (Fig. 4e and Fig. S5). To further validate the binding potential, a dual-luciferase reporter assay was performed. The overexpression of miR-361-3p significantly reduced the luciferase activity of wild-type LINC00922 vector (WT LINC00922) but failed to decrease that of mutant LINC00922 vector (MUT LINC00922), indicating miR-361-3p could directly bind to LINC00922 in SKOV-3 and ES-2 cells (Fig. 4f). While overexpression of miR-361-3p decreased the luciferase activity of wild-type CLDN1 vector (WT CLDN1) but not the mutant CLDN1 vector (MUT CLDN1) (Fig. 4g), indicating that CLDN1 was the direct target of miR-361-3p in SKOV-3 and ES-2 cells. The above results suggested that LINC00922 could serve as a molecular sponge for miR-361-3p and CLDN1 was a direct target gene of miR-361-3p in OC cells.

Hence, we hypothesized LINC00922 could regulate the expression of CLDN1 in OC cells by regulating miR-361-3p. In order to verify this hypothesis, the mRNA and protein expression of CLDN1 were detected in OC cells with sh-linc00922, miR-361-3p inhibitor or sh-linc00922 + miR-361-3p inhibitor, respectively. LINC00922 knockdown markedly increased the miR-361-3p expression in SKOV-3 and ES-2 cells, but silencing miR-361-3p eliminated the promotion effect of LINC00922 knockdown on miR-361-3p expression (Fig. S6). Meanwhile, the LINC00922 knockdown evidently decreased the mRNA and protein expression of CLDN1 in SKOV-3 and ES-2 cells, and silencing miR-361-3p evidently increased the mRNA and protein expression of CLDN1. In the rescue experiments, silencing miR-361-3p could eliminate the corresponding inhibit in the mRNA and protein expression of CLDN1 by LINC00922 knockdown in OC cells (Fig. 4h-j). Taken together, our results indicated that LINC00922 could regulate CLDN1 expression through binding to miR-361-3p in OC cells.

### LINC00922 knockdown inhibits proliferation, migration, invasion and EMT of ovarian cancer cells via regulating miR-361-3p

**Fig. 5:**
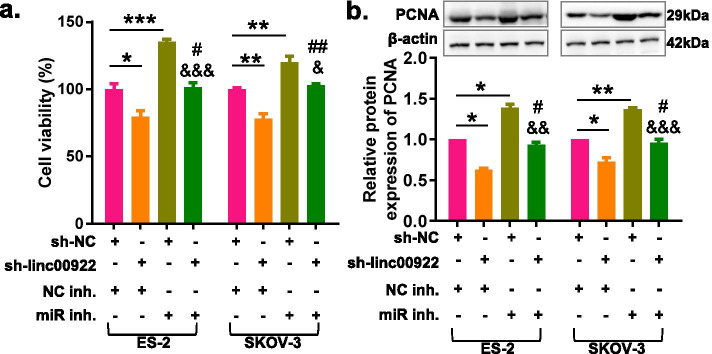
LINC00922 knockdown inhibits proliferation of ovarian cancer cells via regulating miR-361-3p. **a** CCK-8 assay was used to determine the proliferation of OC cells co-transfected with LINC00922 knockdown and silencing miR-361-3p. **b** Western blot assay was used to determine the PCNA protein expression of OC cells co-transfected with LINC00922 knockdown and silencing miR-361-3p. *P < 0.05, **P < 0.01, ***P < 0.001 vs. sh-linc00922 + NC inh. ^#^*P* < 0.05, ^##^*P* < 0.01, ^###^*P* < 0.001 vs. sh-linc00922 + NC inh. ^&&&^*P* < 0.001 vs. sh-NC + miR inhibitor

**Fig. 6:**
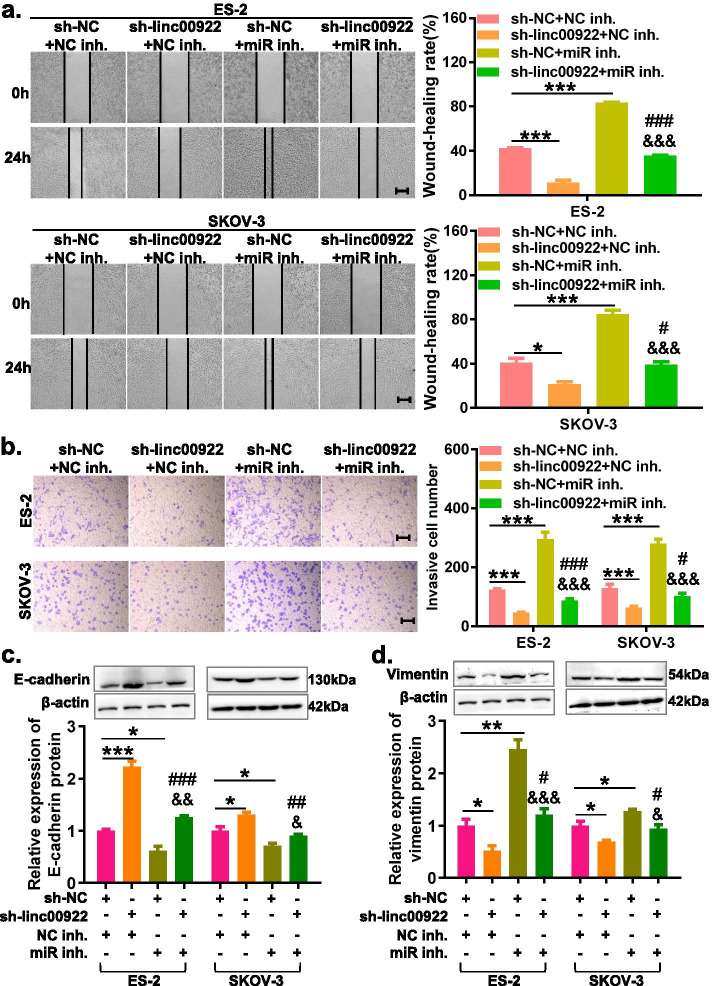
LINC00922 knockdown inhibits migration, invasion and EMT of ovarian cancer cells via regulating miR-361-3p. **a** Wound healing assay was used to determine the migration of ovarian cancer cells co-transfected with LINC00922 knockdown and silencing miR-361-3p. **b** Transwell invasion assay was used to determine the invsaion of ovarian cancer cells co-transfected with LINC00922 knockdown and silencing miR-361-3p. **c**, **d** Western blot assay was used to determine the EMT-related protein expression of ovarian cancer cells co-transfected with LINC00922 knockdown and silencing miR-361-3p. Scale bar: 50 μm. sh-NC, corresponding negative control of sh-linc00922. miR inh., miR-361-3p inhibitor. NC inh., corresponding negative control of miR-361-3p inhibitor. *P < 0.05, **P < 0.01, ***P < 0.001 vs. sh-linc00922 + NC inh. ^#^*P* < 0.05, ^##^*P* < 0.01, ^###^*P* < 0.001 vs. sh-linc00922 + NC inh. ^&^*P* < 0.05, ^&&^*P* < 0.01, ^&&&^*P* < 0.001 vs. sh-NC + miR inhibitor

In order to identify the effect of miR-361-3p over the role of LINC00922 in instigating the oncogenicity in that of OC cells, rescue experiments were designed using miR-361-3p inhibitors. The results indicated that the miR-361-3p inhibitor reversed the proliferation- suppressing effects of LINC00922 knockdown in SKOV-3 and ES-2 cells by CCK-8 assay and western-blot assays (Fig. 5). Moreover, miR-361-3p inhibitor could rescue the migration and invasion suppressing effects of LINC00922 knockdown in SKOV-3 and ES-2 cells by wound healing assay and transwell assays (Fig. 6a, b). Western blot analysis displayed that LINC00922 knockdown significantly increased E-cadherin protein expression and decreased Vimentin protein expression, and these effects could be abolished by miR-361-3p inhibitor (Fig. 6c, d). In addition, we found that silencing miR-361-3p promoted the proliferation, migration, invasion and EMT of SKOV-3 and ES-2 cells (Figs. 5 and 6). These results suggested that miR-361-3p acted as a tumor suppressor in OC, and LINC00922 could promote cell proliferation and invasion by regulating miR-361-3p in OC.

### LINC00922 knockdown inhibits Wnt/β-catenin signaling by upregualting miR-361-3p in ovarian cancer

**Fig. 7:**
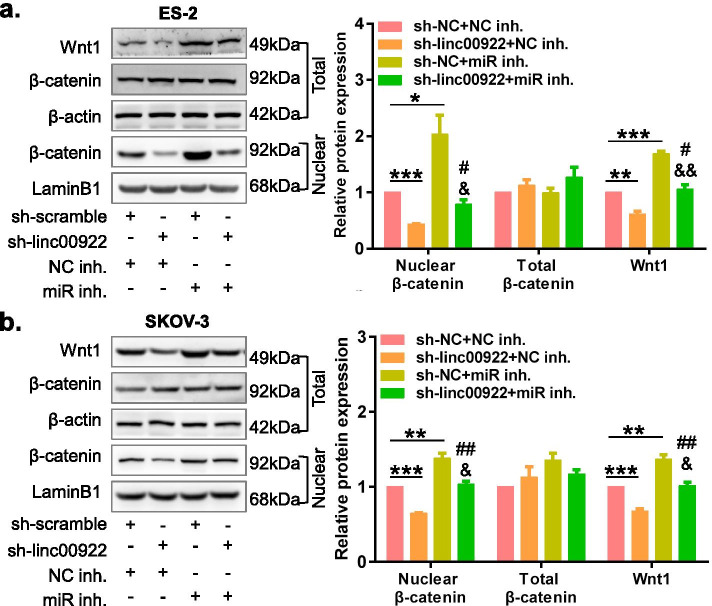
LINC00922 knockdown downregulated Wnt/β-catenin pathway by upregulating miR-361-3p. **a** Western blot assay was used to determine the Wnt/β-catenin-related protein expression of ES-2 cells co-transfected with LINC00922 knockdown and silencing miR-361-3p. **b** Western blot assay was used to determine the Wnt/β-catenin-related protein expression of SKOV-3 cells co-transfected with LINC00922 knockdown and silencing miR-361-3p. sh-linc00922, LINC00922 small hairpin RNA. sh-NC, corresponding negative control of sh-linc00922. miR inh., miR-361-3p inhibitor. NC inh., corresponding negative control of miR-361-3p inhibitor. *P < 0.05, **P < 0.01, ***P < 0.001 vs. sh-linc00922 + NC inh. ^#^*P* < 0.05, ^##^*P* < 0.01 vs. sh-linc00922 + NC inh. ^&^*P* < 0.05, ^&&^*P* < 0.01 vs. sh-NC + miR inhibitor

The oncogenesis of OC is aggravated by the instigation of the Wnt/ β-catenin pathway [19, 20]. Here, we hypothesized that LINC00922 might have triggered tumor progression in that of OC cells by activating the Wnt/β-catenin signaling. For verification of our hypothesis, we carried out co-transfection of ES-2 and SKOV3 cells with that of sh-linc00922 in addition to either miR-321-3p inhibitor or NC inhibitor. Followed by transfection, we detected the expression of β-catenin and Wnt1 protein by western blotting. The results showed that LINC00922 knockdown was significantly decreased the Wnt1 protein expression and β-catenin nuclear expression in SKOV-3 and ES-2 cells compared with those in the control group (Fig. 7), indicating that LINC00922 activated Wnt/β-catenin signaling pathway in ovarian cancer. In addition, silencing miR-361-3p was significantly promoted the Wnt1 protein expression and β-catenin nuclear expression (Fig. 7), indicating that miR-361-3p inhibited Wnt/β-catenin signaling pathway in ovarian cancer**.** In the rescue experiments, silencing miR-361-3p was significantly counteracted the corresponding inhibit the protein expression of Wnt1 and nuclear β-catenin by LINC00922 knockdown in OC cells (Fig. 7). Taken together, these results suggested that LINC00922 promoted OC progression at least up to some extent via activation of the Wnt/β-catenin pathway by down-regulating miR-361-3p expression.

## Discussion

Recently, it has been determined that lncRNAs could perform a promising role in the occurrence and development of many human cancers [21, 22]. The aberrant expression of lncRNAs have been reported in OC, and lncRNAs played an important role in multiple malignancy processes of OC [9, 10, 23]. Therefore, the application of lncRNAs in OC can be promising for the diagnosis, therapy, and prognosis. Although several dysregulated lncRNAs have been identified in OC, more studies are need to elucidate their function and mechanisms. In this study, we designed to identify important lncRNAs involved in the OC progression, and to explore the role and molecular mechanism of LINC00922 in OC.

Upregulation of the LINC00922 was reported in several cancers, including breast cancer, lung cancer and osteosarcoma. It is supposed to be associated with poor clinical results by the targets detected with these cancers [24–27]. It was confirmed from the functional research that LINC00922 promoted cell migration, invasion, and proliferation in breast cancer [24]. Moreover, the latest study identified that LINC00922 regulated EMT in breast cancer through promoting NKD2 methylation [25]. In addition, LINC00922 was acted as a key regulator in the progression of lung cancer [27] and significantly accelerated osteosarcoma doxorubicin(DXR)-resistance [26]. All the above researches indicated that LINC00922 has been shown to play a critical role in many tumor progressions. However, the expression and role of LINC00922 in OC has not been fully conducted. In this study, GEO dataset and TCGA/GTEx dataset were used to explore the LINC00922 expression in OC. Similar to other cancers, our results indicated that the LINC00922 was significantly overexpressed in OC tissues and cells, and upregulated LINC00922 had significantly positive correlation with poor prognosis of OC patients. Functionally, LINC00922 knockdown blocked OC cells proliferation, invasion, migration and EMT in vitro and tumor growth in vivo.

Previously, increasing researches have also shown that the functions of lncRNAs are closely associated with their subcellular localization [28, 29]. In this study, the LINC00922 distribution in OC cells was evaluated by using the nuclear-cytoplasmic fractionation and RNA FISH assays. The results showed that LINC00922 was mainly localized in cytoplasm, indicating its potential for functioning as a miRNA sponge. Some latest studies have been proposed the hypothesis of ceRNA, which suggested that lncRNAs comprised of miRNA responsive elements [30, 31]. These elements function as a molecular sponge of miRNAs in human-related cancers, in such a way they protect mRNAs from their suppression and degradation [32]. Such crosstalk between the lncRNA and miRNA regulates the expression of genes and therefore pathological processes happen to be induced during cancer progression and genesis [33, 34]. Subsequently, we constructed a new LINC00922/miR-361-3p/CLDN1 triple sub-network based on the comprehensive analysis of the data miming and bioinformatics analysis.

The analysis indicated that there existed binding sites of miR-361-3p in the LINC00922 or CLDN1, which was further validated by dual-luciferase reporter assay. Previously, many studies reported miR-361-3p overexpression in human-related cancers such as lung cancer [35]. The anti-oncogenic role of miR-361-3p was proved and has also shown the ability to suppress ovarian cancer progression [36]. Consistent with former studies, we also found that miR-361-3p silencing promote malignant phenotype of OC cells, which was rescued by simultaneous LINC00922 knockdown. And we also found that miR-361-3p targeted binding CLDN1 3’UTR to inhibit CLDN1 expression. CLDN1 (Claudin-1) is considered as major constituents of the tight junction complexes, which was expressed in almost all known types of epithelial cells and it plays a major role in the regulation of intercellular permeability [37]. Studies have showed that CLDN1 was a double-edged sword in cancer [38]. For example, CLDN1 knockdown promoted tumor growth and metastases in pancreatic cancer [39] and lung cancer [40] both in vitro and in vivo, indicating CLDN1 could act as a suppressor. In addition, studies have shown that CLDN1 played an important oncogene in many cancers, such as esophageal squamous carcinoma [41], hepatocellular carcinoma [42] and colorectal cancer [43]. Importantly, previous studies indicated that reduced CLDN1 expression could inhibited ovarian cancer cells mobility and invasion in vitro and tumor growth in vivo [44, 45]. Another study reported CLDN1 was mainly expressed in ovarian epithelial tumors for indication of epithelial differentiation [46]. However, the molecular mechanisms underlying the dysregulation of CLDN1 in OC remain unclear. Our results indicated that CLDN1 was upregulated in OC tissues and cells. Interestingly, our findings suggested that the abnormal high expression of CLDN1 was regulated by the LINC00922/miR-361-3p axis in OC.

The Wnt/β-catenin signaling pathway exerted tumor-promoting role in OC, and is recognized as an important candidate for targeted therapies of the OC development [47]. Moreover, multiple non-coding RNAs (ncRNAs) have been reported to regulate OC progression through the modulation of Wnt/β-catenin signaling pathway [48–50]. It was been reported that lncRNA KCNQ1OT1 promoted the β-catenin expression and thus facilitated the proliferation and migration of OC cells [51]. The miR-16 inhibited the migration and invasion of OC cells via inactivation of the Wnt/β-catenin signaling pathway [52]. LncRNA HOXD-AS1 promoted the cell proliferation, invasion and epithelial-mesenchymal transition through sponging miR-133a-3p and activating Wnt/β-catenin signaling pathway in epithelial ovarian cancer [53]. LncRNA BLACAT1 knockdown inhibited the ovarian cancer progression via suppressing the Wnt/β-catenin signaling pathway by sponging miR-519b-3p [54]. However, it is unclear whether LINC00922/miR-361-3p axis regulates the Wnt/β-catenin signaling pathway. In this study, we found silencing miR-361-3p in OC cells increased the protein expression of Wnt1 and nuclear β-catenin but not total β-catenin, which suggested that Wnt/β-catenin signaling pathway was activated by silencing miR-361-3p. Meanwhile, we also found that LINC00922 knockdown inhibited Wnt/β-catenin signaling pathway via negatively regulating miR-361-3p expression. Studies have demonstrated that abnormal activation of Wnt/β-catenin signaling pathway led to the nuclear accumulation of β-catenin to promote cells proliferation, migration, invasion and EMT, thereby promoting tumorgenicity and metastasis of tumor [55–57]. Increasing studies in OC have shown that inhibition of this signaling pathway can strongly inhibit tumor growth and metastasis [58, 59]. Consequently, we speculated that LINC00922/miR-361-3p axis might regulate the proliferation, migration, invasion and EMT of OC cells through the activation of Wnt/β-catenin signaling pathway. However, the specific regulatory mechanism of LINC00922/miR-361-3p axis regulating the Wnt/β-catenin signaling pathway remains to be further investigated.

## Conclusion

In conclusion, the present study provides direct evidence that LINC00922 contributes OC progression through upregulating CLDN1 and activating Wnt/β-catenin by inhibiting miR-361-3p, suggesting LINC00922 might be a novel therapeutic target for OC intervention.

## Supplementary Information

**Additional file 1:** **Fig. S1.** Identification of differential expression of LncRNAs in OC tissues. (a) Principal component analysis of lncRNAs based on OC microarray data (GSE74448). Blue dots represent normal ovarian tissue samples, pink dots represent OC tissue samples. (b) Volcano plot depicts differential expression of LncRNAs between normal and OC tissue samples. The upregulation and downregulation of genes are displayed respectively in red and green. Values are demonstrated as the log_2_ of tag counts. (c) Heatmap showing differentially expressed LncRNAs based on OC microarray data (GSE74448). Each lane resembles an independent biological sample.

**Additional file 2:** **Fig. S2.** Differentially expressed LncRNAs with survival in OC were analyzed based on G74448 and TCGA data sets. (a) The expression level of DANT2, LINC001711, and SPATA41 in that of OC tissues as well as normal ovarian tissues. (b) Kaplan-Meier curve was plotted for the analysis of patients' survival rate on the basis of expression of DANT2, LINC001711, as well as SPATA41. (c) RT-qPCR was used to quantify DANT2, LINC001711, and SPATA41 expression in three OC cell lines namely A2780, SKOV3, and ES-2 in addition to an ovarian epithelial cell line of human named IOSE-80. ^#^*P *< 0.05; ^##^*P *< 0.01; ^###^*P *< 0.001.

**Additional file 3:** **Fig. S3.** The transfection effect of LINC00922 shRNA in OC cells was evaluated by RT-qPCR. Sh-linc00922-3 was selected for further experiments.**P < 0.01; ***P < 0.001.

**Additional file 4: Fig. S4.** The LINC00922-miRNA-mRNA ceRNA network in OC. (a) Venn diagram depicting the convergence between LINC00922 target miRNAs and differentially downregulated miRNAs in microarray data GSE119055. (b) Venn diagram depicting the convergence between predicted targets of 11 miRNAs and differentially upregulated mRNAs in microarray data GSE74448. (c) The network comprising LINC00922, 11 miRNAs, along with 307 genes generated with Cytoscape v.3.6.1. (d) Correlations between LINC00922 and miRNAs, miRNAs and mRNA, LINC00922 and mRNAs (only significant correlation is shown). (e) Schematic diagram of LINC00922 /miR-361-3/CLDN1 embroiled in the regulation mechanism of OC progression.

**Additional file 5:** **Fig. S5.** The protein expression of CLDN1 were detected by western blotting in ES-2 and SKOV-3 cells transfected with either miR-361-3p mimics or miR-361-3p inhibitor. miR mim., miR-361-3p mimics. NC mim., corresponding negative control of miR-361-3p mimics. miR inh., miR-361-3p inhibitor. NC inh., corresponding negative control of miR-361-3p inhibitor. **P < 0.01, ***P < 0.001.

**Additional file 6:** **Fig. S6.** Alterations in the expression levels of miR-361-3p were identified in ES-2 and SKOV-3 cells after passing through co-transfection with sh-linc00922 along with either miR-361-3p inhibitor or NC inhibitor. sh-linc00922, LINC00922 small hairpin RNA. sh-NC, corresponding negative control of sh-linc00922. miR inh., miR-361-3p inhibitor. NC inh., corresponding negative control of miR-361-3p inhibitor. *P < 0.05, **P < 0.01, ***P < 0.001 vs. sh-linc00922 + NC inh. ^###^*P*<0.001 vs. sh-linc00922+NC inh. ^&&&^*P*<0.001 vs. sh-NC + miR inhibitor.

## Data Availability

The datasets generated and analyzed during the current study are available from TCGA and GEO database.
