# Supplementary figures and images for: The LINC00922 aggravates ovarian cancer progression via sponging miR-361-3p
Source: J Ovarian Res. 2021 Jun 11;14:77. doi: 10.1186/s13048-021-00828-7 (PMC8194245; doi:10.1186/s13048-021-00828-7)

**a.**

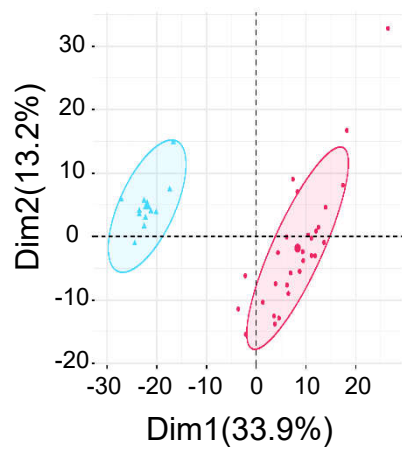

**b.**

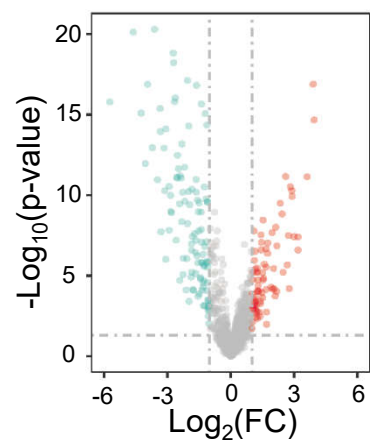

**c.**

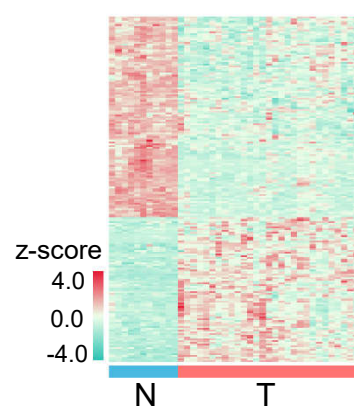

Supplement: Supplementary file 1 — Additional file 1: Fig. S1. Identification of differential expression of LncRNAs in OC tissues. (a) Principal component analysis of lncRNAs based on OC microarray data (GSE74448). Blue dots represent normal ovarian tissue samples, pink dots represent OC tissue samples. (b) Volcano plot depicts differential expression of LncRNAs between normal and OC tissue samples. The upregulation and downregulation of genes are displayed respectively in red and green. Values are demonstrated as the log2 of tag counts. (c) Heatmap showing differentially expressed LncRNAs based on OC microarray data (GSE74448). Each lane resembles an independent biological sample. [file 13048_2021_828_MOESM1_ESM.pdf]

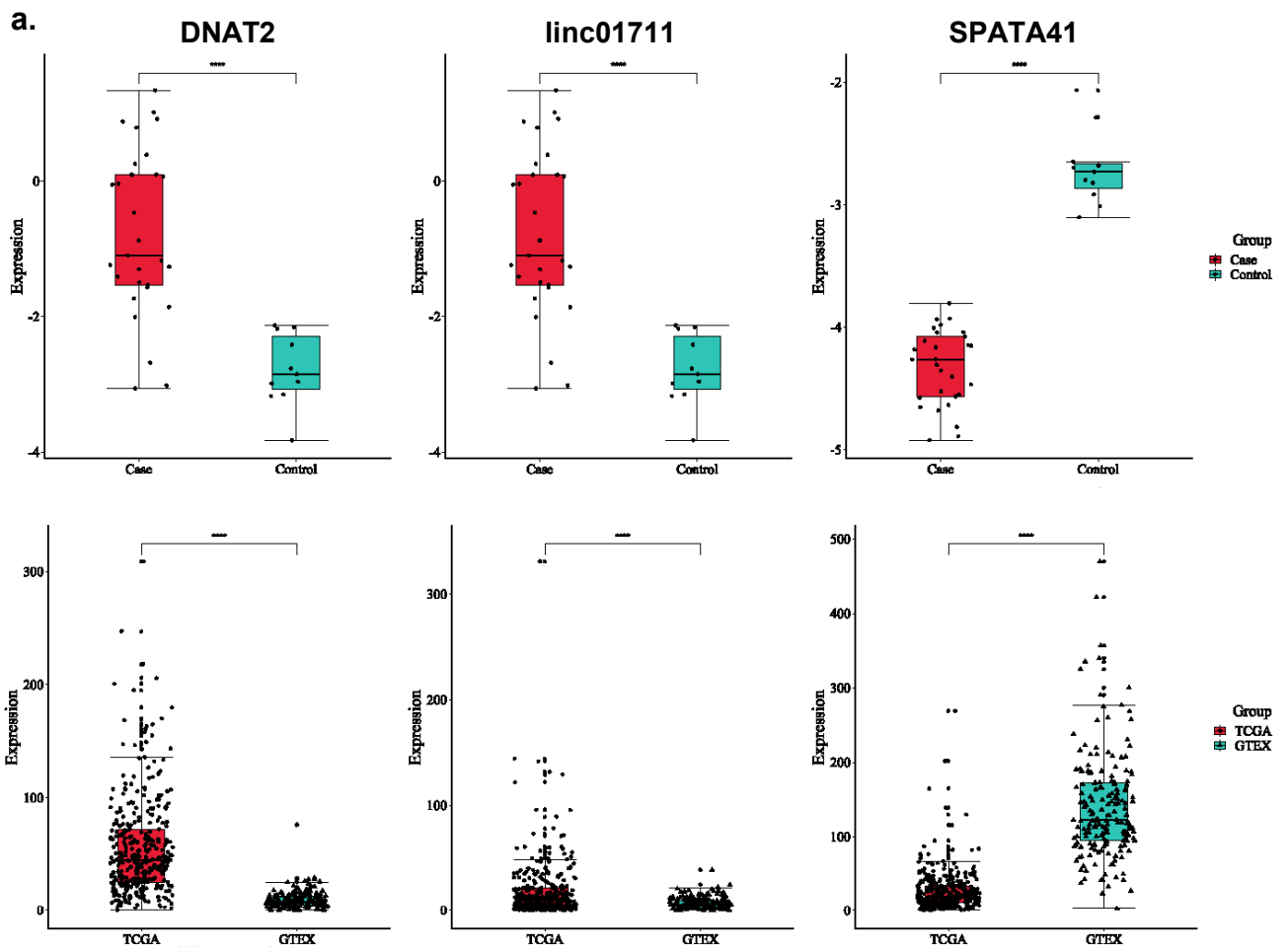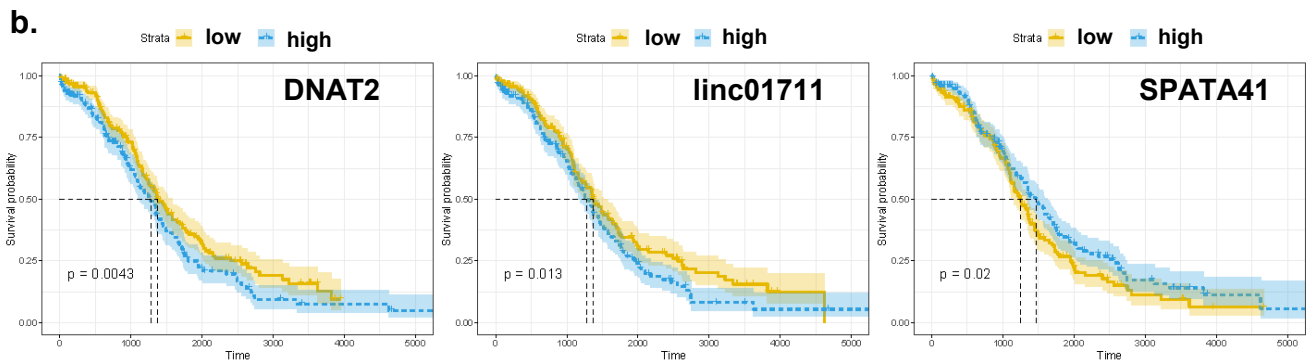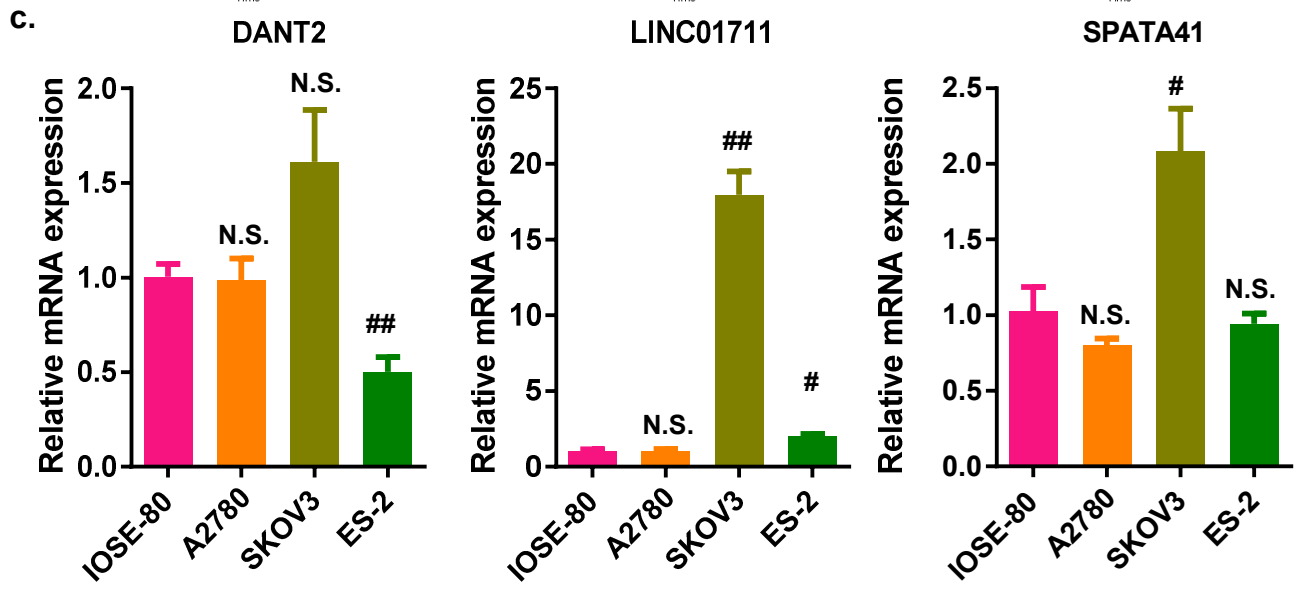

Supplement: Supplementary file 2 — Additional file 2: Fig. S2. Differentially expressed LncRNAs with survival in OC were analyzed based on G74448 and TCGA data sets. (a) The expression level of DANT2, LINC001711, and SPATA41 in that of OC tissues as well as normal ovarian tissues. (b) Kaplan-Meier curve was plotted for the analysis of patients' survival rate on the basis of expression of DANT2, LINC001711, as well as SPATA41. (c) RT-qPCR was used to quantify DANT2, LINC001711, and SPATA41 expression in three OC cell lines namely A2780, SKOV3, and ES-2 in addition to an ovarian epithelial cell line of human named IOSE-80. #P < 0.05; ##P < 0.01; ###P < 0.001. [file 13048_2021_828_MOESM2_ESM.pdf]

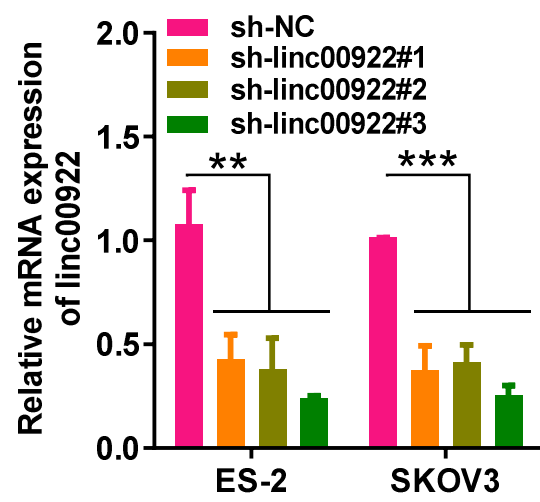

Supplement: Supplementary file 3 — Additional file 3: Fig. S3. The transfection effect of LINC00922 shRNA in OC cells was evaluated by RT-qPCR. Sh-linc00922-3 was selected for further experiments.**P < 0.01; ***P < 0.001. [file 13048_2021_828_MOESM3_ESM.pdf]

**a. Potential binding of miRNAs to *linc00922***

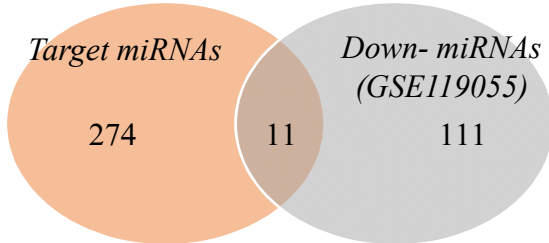

**b. Potential target genes of the miRNAs**

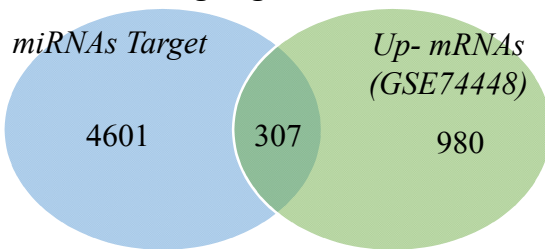

**c.**

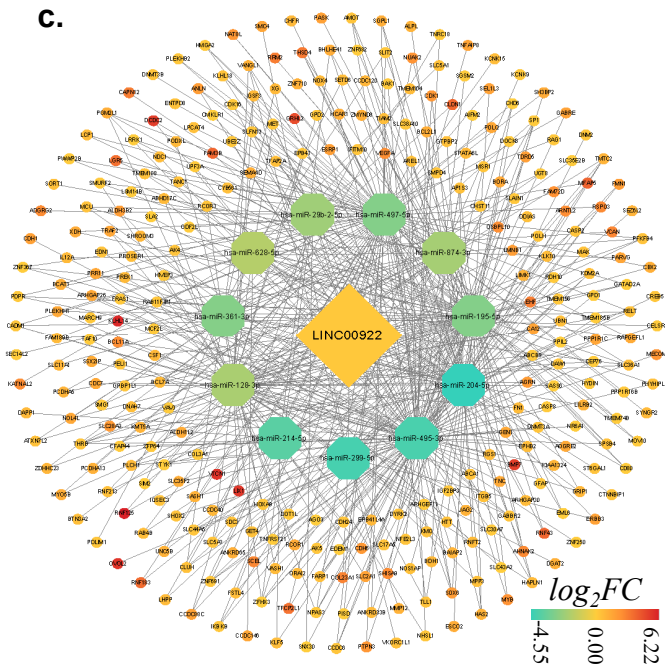

**d.**

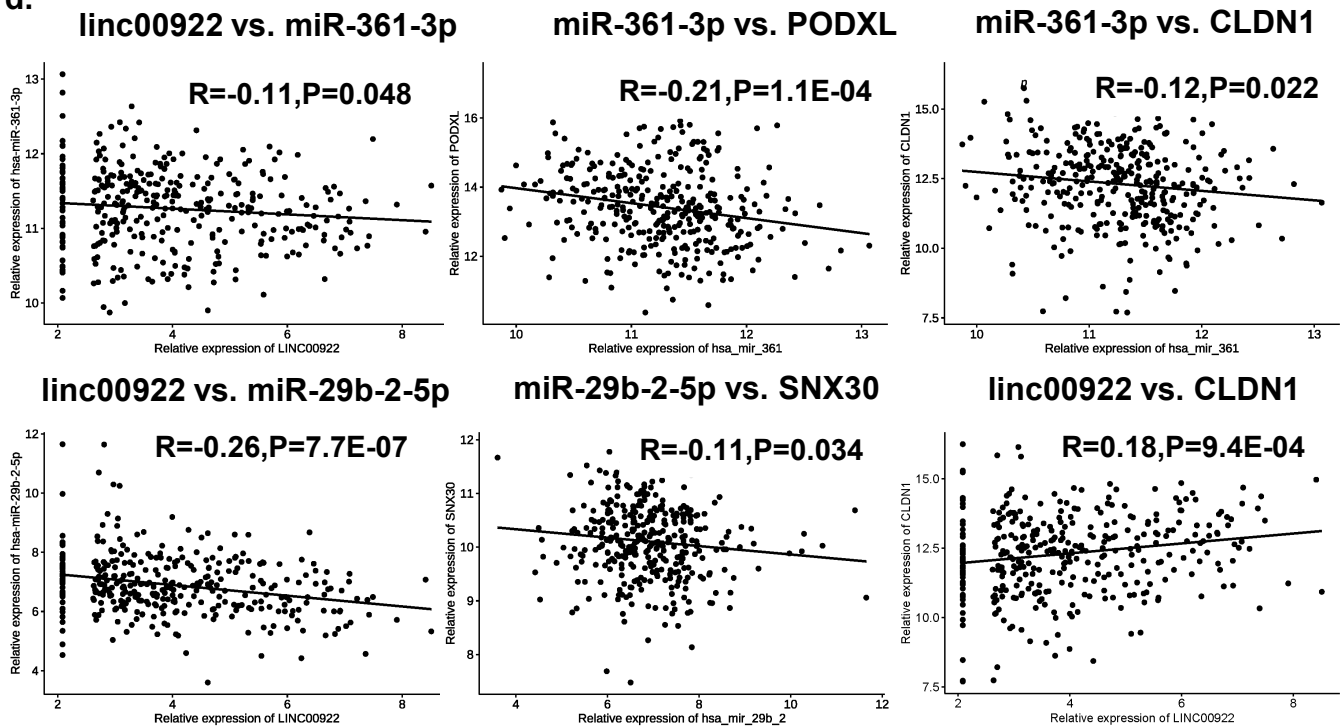

**e.**

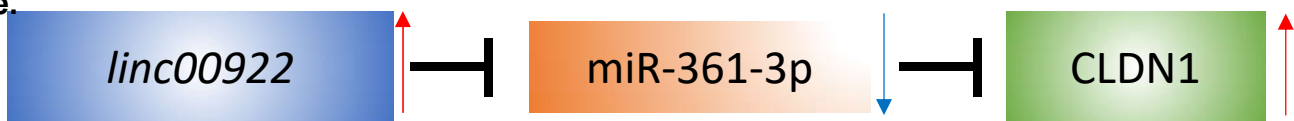

Supplement: Supplementary file 4 — Additional file 4: Fig. S4. The LINC00922-miRNA-mRNA ceRNA network in OC. (a) Venn diagram depicting the convergence between LINC00922 target miRNAs and differentially downregulated miRNAs in microarray data GSE119055. (b) Venn diagram depicting the convergence between predicted targets of 11 miRNAs and differentially upregulated mRNAs in microarray data GSE74448. (c) The network comprising LINC00922, 11 miRNAs, along with 307 genes generated with Cytoscape v.3.6.1. (d) Correlations between LINC00922 and miRNAs, miRNAs and mRNA, LINC00922 and mRNAs (only significant correlation is shown). (e) Schematic diagram of LINC00922 /miR-361-3/CLDN1 embroiled in the regulation mechanism of OC progression. [file 13048_2021_828_MOESM4_ESM.pdf]

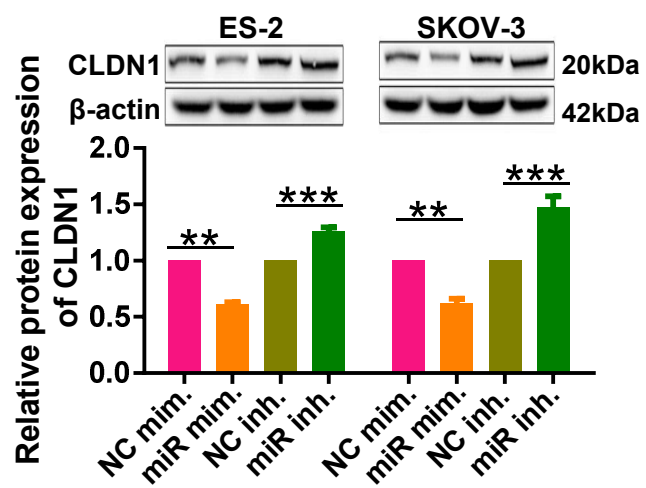

Supplement: Supplementary file 5 — Additional file 5: Fig. S5. The protein expression of CLDN1 were detected by western blotting in ES-2 and SKOV-3 cells transfected with either miR-361-3p mimics or miR-361-3p inhibitor. miR mim., miR-361-3p mimics. NC mim., corresponding negative control of miR-361-3p mimics. miR inh., miR-361-3p inhibitor. NC inh., corresponding negative control of miR-361-3p inhibitor. **P < 0.01, ***P < 0.001. [file 13048_2021_828_MOESM5_ESM.pdf]

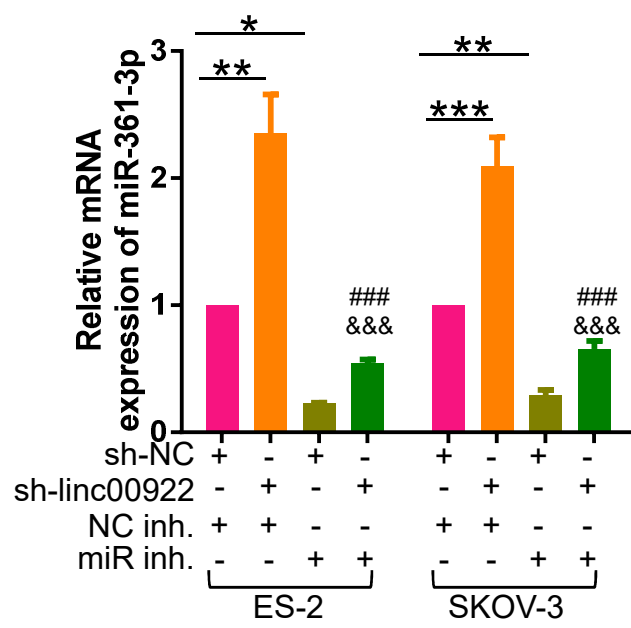

Supplement: Supplementary file 6 — Additional file 6: Fig. S6. Alterations in the expression levels of miR-361-3p were identified in ES-2 and SKOV-3 cells after passing through co-transfection with sh-linc00922 along with either miR-361-3p inhibitor or NC inhibitor. sh-linc00922, LINC00922 small hairpin RNA. sh-NC, corresponding negative control of sh-linc00922. miR inh., miR-361-3p inhibitor. NC inh., corresponding negative control of miR-361-3p inhibitor. *P < 0.05, **P < 0.01, ***P < 0.001 vs. sh-linc00922 + NC inh. ###P<0.001 vs. sh-linc00922+NC inh. &&&P<0.001 vs. sh-NC + miR inhibitor. [file 13048_2021_828_MOESM6_ESM.pdf]
